# Beyond Xerostomia: A Two-Year Follow-Up Case Report of Periodontal and Peri-Implant Health in Primary Sjögren Syndrome

**DOI:** 10.7759/cureus.117413

**Published:** 2026-10-04

**Authors:** Joey Nammour, Elsa Boustany, Samar Bou Jaoude, Ghassan Yared, Wassim Manhal

**Affiliations:** 1 Faculty of Dental Medicine, Saint Joseph University of Beirut, Beirut, LBN; 2 Department of Oral Medicine and Radiology, Saint Joseph University of Beirut, Beirut, LBN; 3 Cranio-Facial Research Laboratory, Department of Oral Pathology, Saint Joseph University of Beirut, Beirut, LBN

**Keywords:** dental implants, periodontal status, primary sjögren’s syndrome, sjögren’s syndrome, systemic disease, xerostomia

## Abstract

Primary Sjögren syndrome (pSS) is an autoimmune disorder characterized by chronic salivary gland dysfunction that predisposes patients to oral complications and tooth loss. Although dental implant therapy has demonstrated favorable survival rates in patients with pSS, evidence regarding long-term peri-implant soft tissue health remains limited. This report describes the diagnosis of pSS following implant rehabilitation and highlights the impact of the disease on peri-implant health during a two-year follow-up. A 61-year-old man underwent implant placement at sites 14 and 16 with simultaneous sinus floor elevation and connective tissue grafting. Several months later, he developed progressive xerostomia, xerophthalmia, burning mouth symptoms, and dysphagia. Histopathological examination of a minor labial salivary gland biopsy revealed focal lymphocytic sialadenitis (Chisholm and Mason grade 3), with findings considered compatible with Sjögren syndrome in the clinical context. Following rheumatologic management with hydroxychloroquine and pilocarpine, the patient was monitored for two years. Clinical examination demonstrated persistent xerostomia and generalized gingival and peri-implant mucosal inflammation, whereas radiographic evaluation showed stable peri-implant bone levels without marginal bone loss. This case supports current evidence indicating that implant osseointegration and short-term survival can be maintained in patients with pSS despite chronic hyposalivation. However, it also illustrates that persistent salivary dysfunction and immune dysregulation may predispose peri-implant tissues to mucosal inflammation in the absence of radiographic bone loss, emphasizing that peri-implant tissue health should be evaluated independently of implant survival. Dental implants appear to represent a reliable rehabilitation option for patients with pSS when combined with individualized supportive care. Nevertheless, long-term success should be assessed not only by implant survival and marginal bone stability but also by careful monitoring of peri-implant soft tissue health. Early recognition of Sjögren syndrome by dental practitioners and multidisciplinary management are essential for optimizing both systemic and implant-related outcomes.

## Introduction

Dental implant therapy has become a predictable and widely accepted treatment for the rehabilitation of partially and completely edentulous patients, demonstrating high long-term survival rates in healthy individuals. However, systemic conditions that alter the oral environment, particularly autoimmune diseases associated with salivary gland dysfunction, continue to raise concerns regarding peri-implant tissue health and the long-term stability of implant-supported rehabilitation [1].

Primary Sjögren syndrome (pSS) is a chronic autoimmune disease characterized by lymphocytic infiltration of the exocrine glands, primarily affecting the salivary and lacrimal glands and resulting in xerostomia and xerophthalmia. Reduced salivary secretion alters both the quantity and composition of saliva, impairing lubrication, antimicrobial defense, buffering capacity, and mechanical clearance of microorganisms. These changes promote oral dysbiosis and increase the risk of dental caries, periodontal disease, oral candidiasis, and other inflammatory oral manifestations. Consequently, many patients with pSS require prosthetic rehabilitation, including dental implants, because of premature tooth loss and poor tolerance of removable prostheses in the severely xerostomic oral environment [2].

Although accumulating evidence suggests that implant therapy can be successfully performed in patients with pSS, most published studies have primarily evaluated implant survival, marginal bone loss, and osseointegration [3]. In contrast, comparatively little attention has been paid to peri-implant soft tissue health and the biological mechanisms that may influence peri-implant tissue homeostasis in these patients. Chronic hyposalivation, alterations in salivary composition, oral microbial dysbiosis, and persistent immune dysregulation may predispose peri-implant tissues to chronic inflammation despite successful osseointegration. Consequently, peri-implant tissue health should be evaluated alongside implant survival, as both parameters are essential for determining the long-term success of implant therapy in patients with pSS.

Dentists are often the first healthcare professionals to evaluate patients presenting with persistent xerostomia and its oral manifestations. Early recognition of Sjögren syndrome is therefore essential not only to establish an accurate diagnosis and initiate multidisciplinary management but also to optimize long-term oral rehabilitation and supportive peri-implant care.

The aim of this case report was to present the diagnosis of pSS following implant rehabilitation and to evaluate peri-implant tissue health over a two-year follow-up period. By comparing the clinical findings with the current literature, this report highlights that implant success in patients with pSS should be assessed not only by implant survival and marginal bone stability but also by careful evaluation of peri-implant soft tissue health and individualized long-term maintenance.

## Case presentation

Patient information and initial dental management

A 61-year-old man presented to the Dental Center of the Faculty of Dental Medicine of Saint Joseph University of Beirut seeking dental implant rehabilitation. He underwent placement of two dental implants at sites 14 and 16, combined with internal sinus floor elevation at site 16 and a connective tissue graft at site 14. The postoperative periapical radiograph obtained following implant placement is presented in Figure 1.

**Figure 1:**
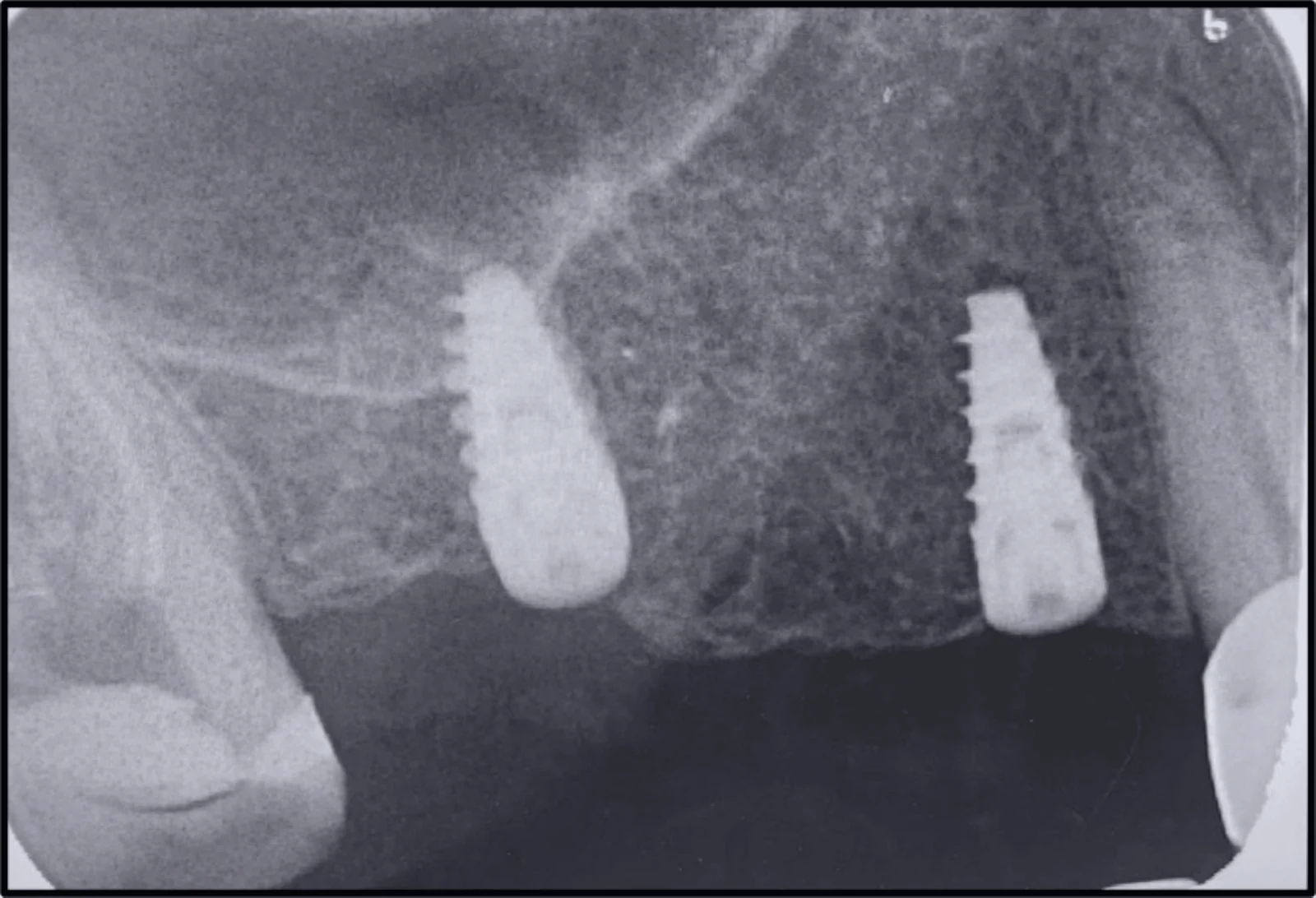
Postoperative periapical radiograph showing dental implants placed at sites 14 and 16.

Clinical presentation and symptoms

In the same year, he began experiencing xerostomia and xerophthalmia. These symptoms were accompanied by burning mouth symptoms, ocular burning, and a sensation of sand in his eyes. To alleviate these symptoms, he increased his water intake and used lubricating eye drops. Six months later, his symptoms had progressively worsened, significantly affecting his quality of life.

Diagnostic assessment

As his symptoms progressively worsened, he was referred by his dentist to the Department of Oral Pathology and Diagnosis at the Faculty of Dental Medicine of Saint Joseph University of Beirut. Given the combination of xerostomia and xerophthalmia, consistent with sicca syndrome (dry syndrome), a minor salivary gland biopsy of the lower lip was performed to investigate the underlying cause. Representative clinical photographs of the biopsy procedure are shown in Figures 2-4.

**Figure 2:**
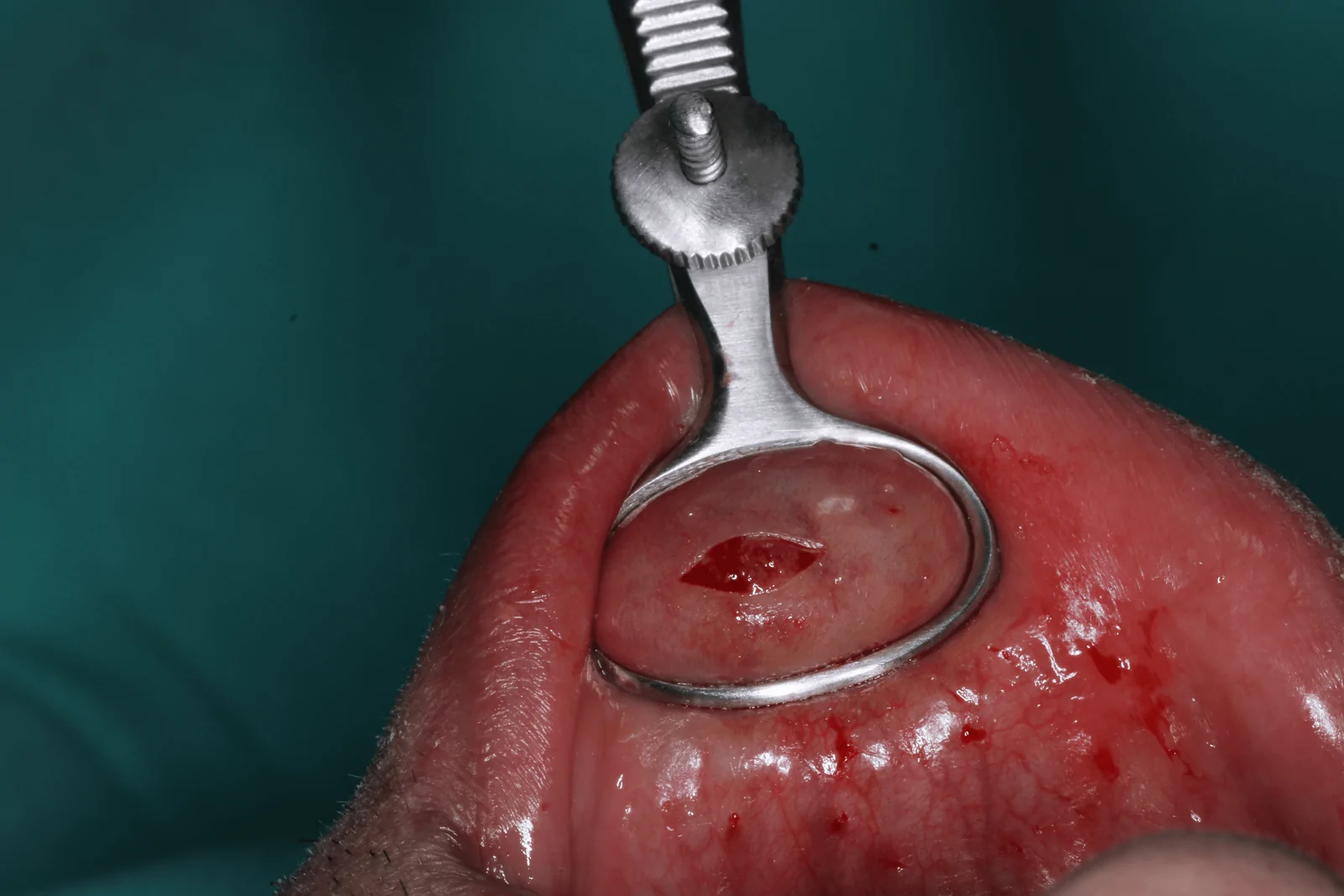
Sequential steps of the minor salivary gland biopsy procedure: initial mucosal incision of the lower lip.

**Figure 3:**
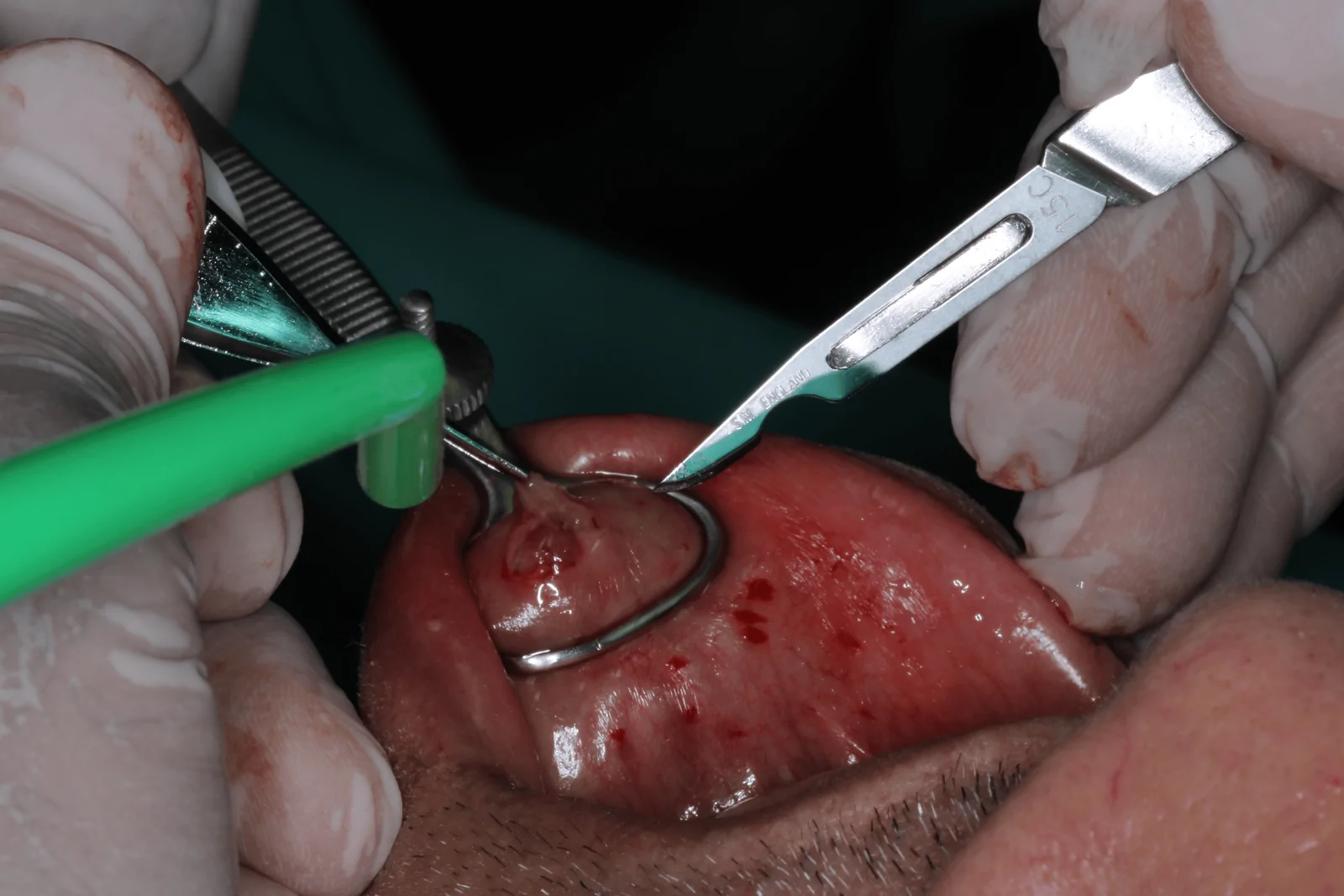
Excision of the minor salivary glands.

**Figure 4:**
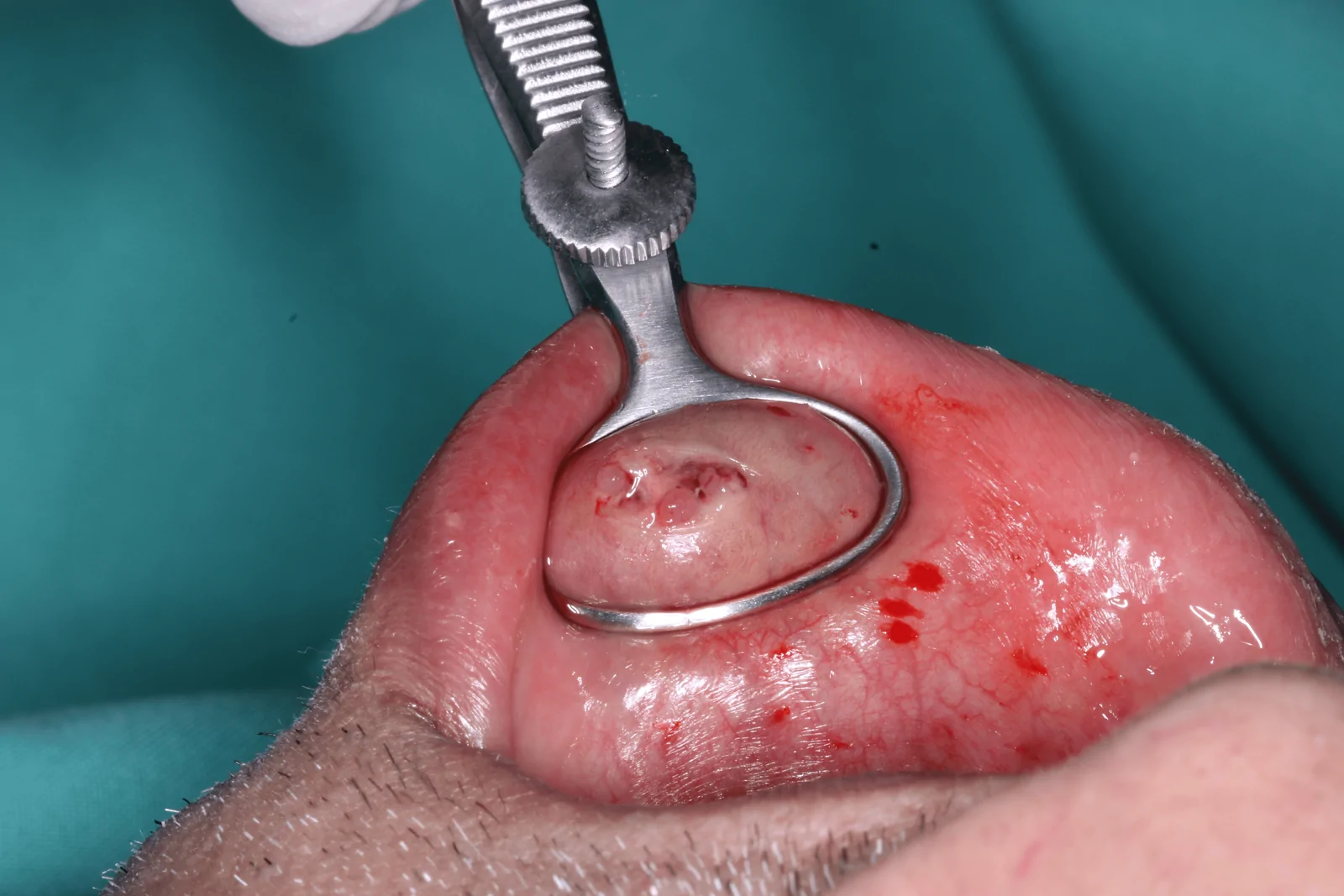
Surgical site after biopsy.

Histopathological findings

The biopsy specimen was subsequently submitted to the National Institute of Pathology, Lebanon, for histopathological examination. Microscopic examination revealed two salivary lobules and a pericanal lymphocytic focus, with slight atrophy of the adjacent ducts and acini. The remaining salivary tissue was unremarkable. The biopsy findings were consistent with focal lymphocytic sialadenitis (Chisholm and Mason grade 3) and were considered compatible with Sjögren syndrome. The diagnosis of pSS was subsequently established following multidisciplinary clinical evaluation. The histopathological assessment was based on the original pathology report; histopathological images were not available for inclusion.

Therapeutic management

The patient was subsequently referred to a rheumatologist, who initiated treatment to alleviate the symptoms of pSS. The prescribed medications included hydroxychloroquine (Dolquine), 200 mg twice daily, an immunomodulatory agent used to reduce autoimmune activity [4], and pilocarpine (Salagen), 5 mg three times daily, prescribed to stimulate salivary secretion and relieve xerostomia [5].

Although these medications do not cure the disease, they help alleviate symptoms and modulate the underlying autoimmune process. A multidisciplinary management approach was implemented to reduce the risk of systemic complications associated with pSS, including pulmonary, renal, and lymphatic involvement.

Follow-up and outcome

At the two-year follow-up, the patient reported persistent symptoms despite treatment. He continued to experience burning mouth symptoms, dysphagia, and intermittent oral pain (stomatodynia). Intraoral examination revealed halitosis, gingival inflammation, and generalized dryness of the oral mucosa. At the two-year follow-up examination, a localized soft tissue alteration was observed in the marginal gingiva of tooth #22, without sufficient clinical findings to establish a specific diagnosis (Figures 5, 6). Clinical examination also revealed peri-implant mucosal inflammation, whereas radiographic evaluation demonstrated no evidence of marginal bone loss around the implants placed at sites 14 and 16 (Figure 7).

**Figure 5:**
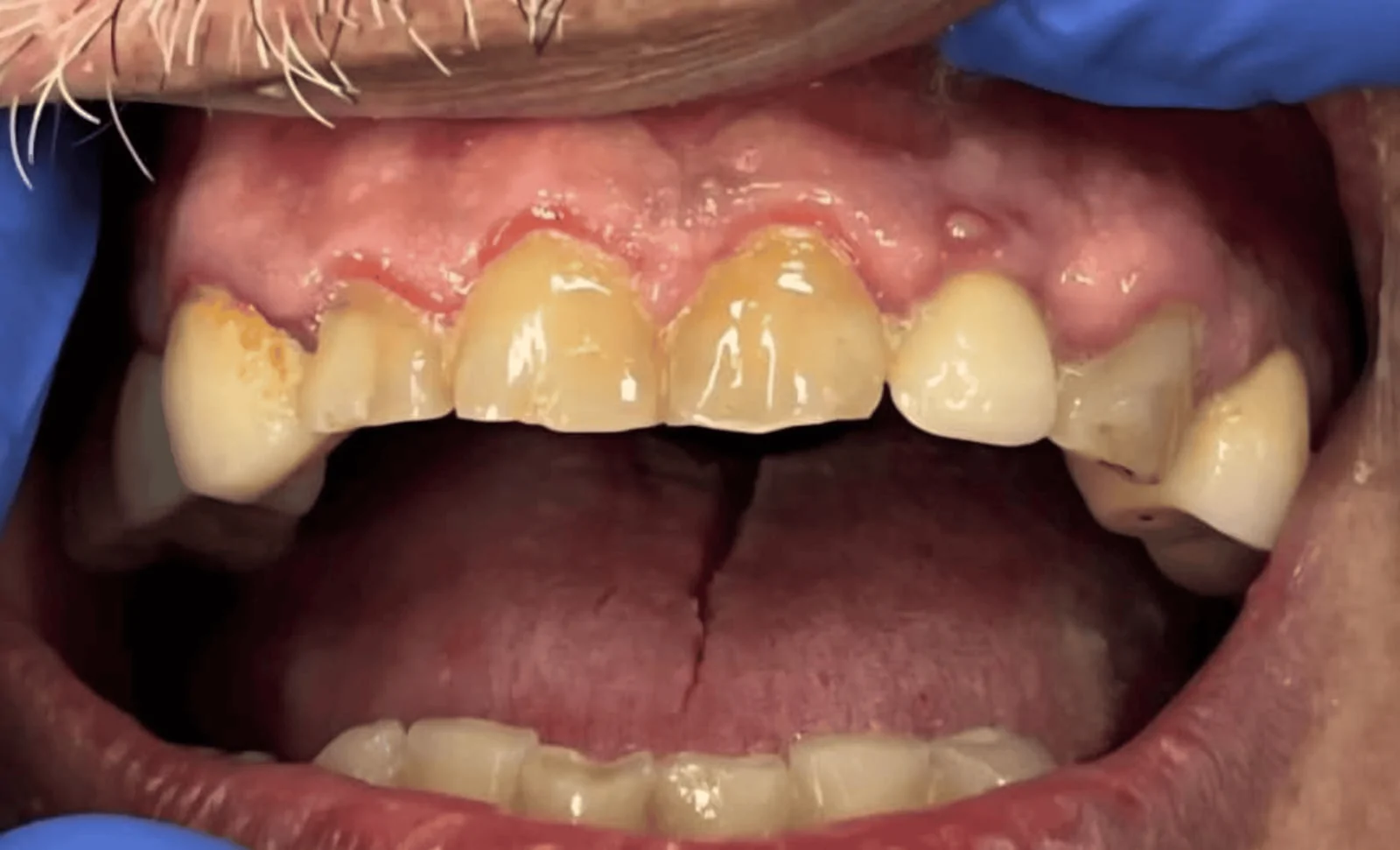
Intraoral clinical photograph at the two-year follow-up showing the maxillary arch with gingival inflammation.

**Figure 6:**
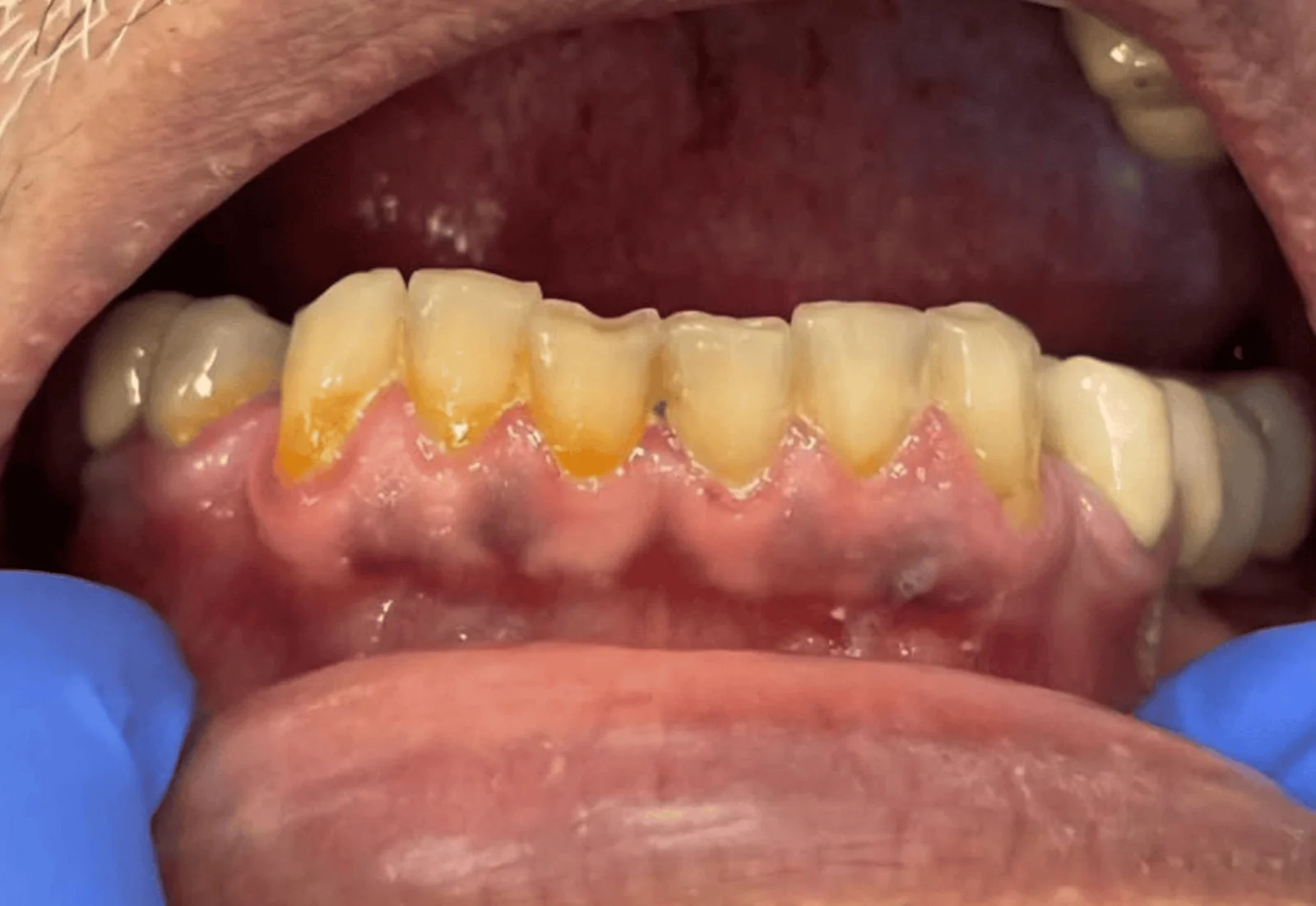
Intraoral clinical photograph at the two-year follow-up showing the mandibular arch with gingival inflammation.

**Figure 7:**
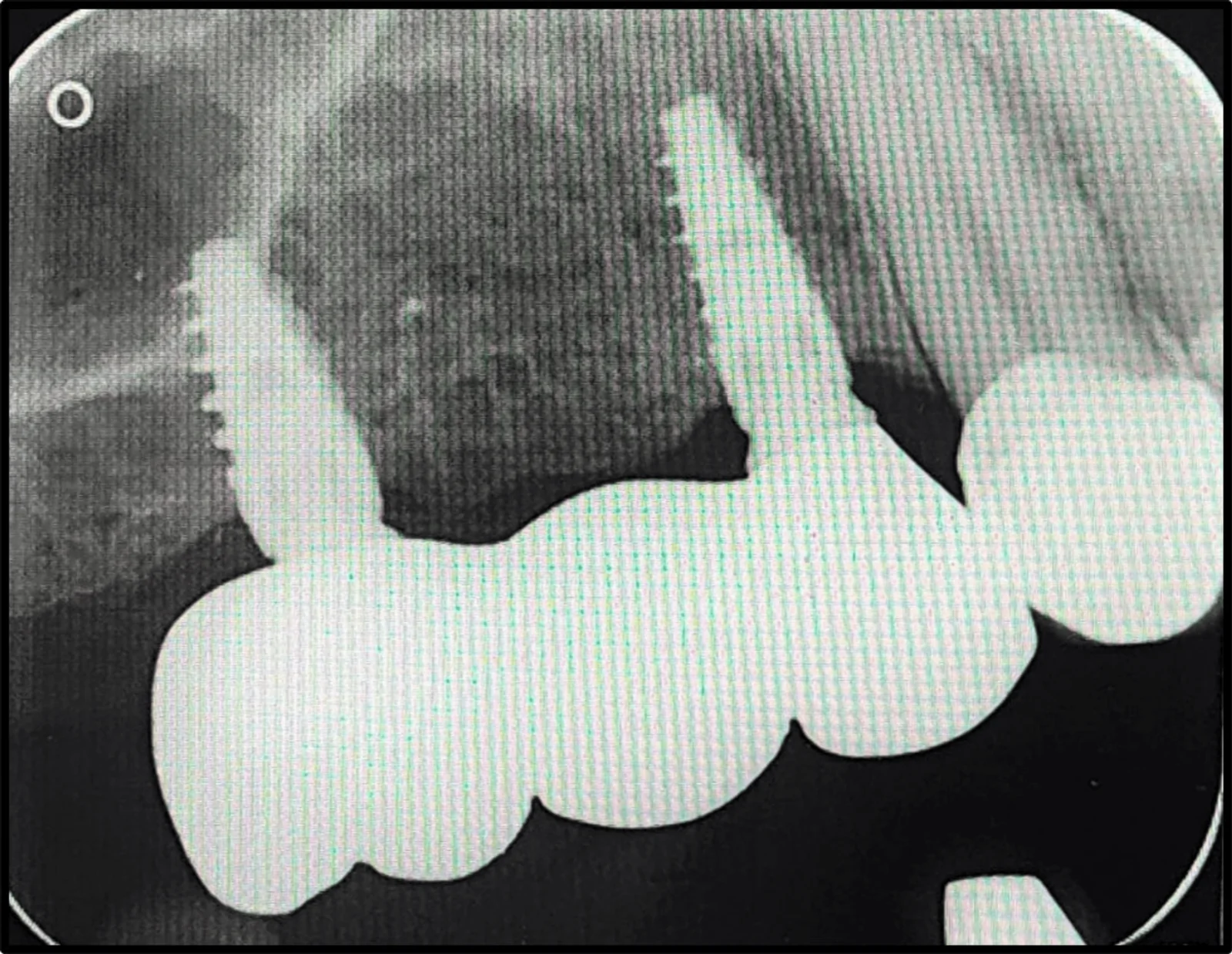
Follow-up periapical radiograph obtained two years after implant placement at sites 14 and 16.

During the documented follow-up visits, the patient’s periodontal and peri-implant conditions were clinically assessed, including evaluation of plaque accumulation and the peri-implant soft tissues.

Dryness of the skin, particularly in the perinasal region, was also observed (Figure 8).

**Figure 8:**
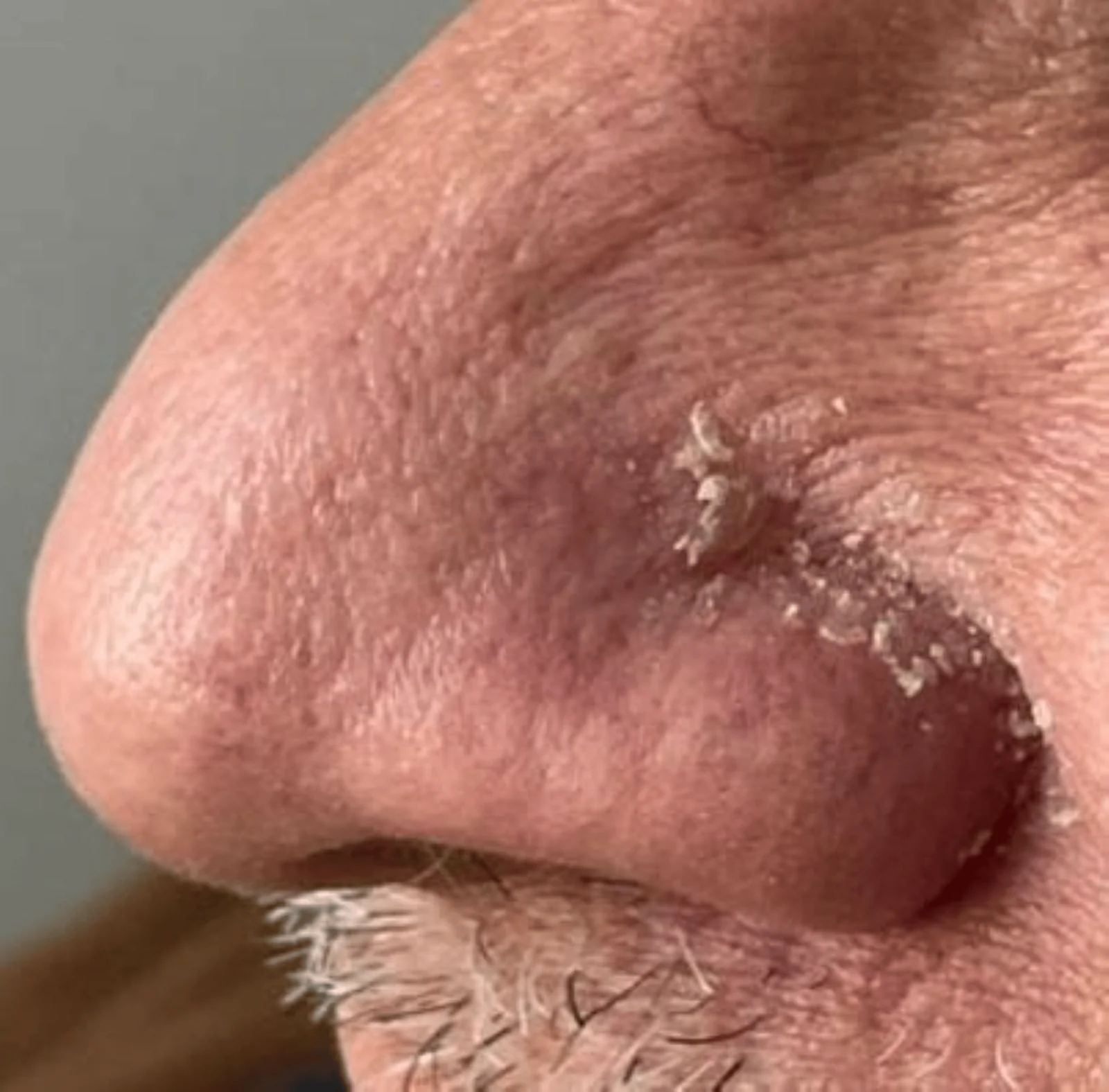
Clinical photograph demonstrating perinasal skin dryness.

According to the patient, the disease had remained stable without noticeable clinical improvement. He also reported persistent fatigue and excessive sleepiness. No clinical manifestations suggestive of secondary autoimmune disease were identified, and the diagnosis remained consistent with pSS.

## Discussion

pSS and oral implications

Sjögren syndrome is a chronic autoimmune disorder affecting the salivary and lacrimal glands, with xerostomia and xerophthalmia representing its characteristic manifestations. This syndrome results from lymphocytic infiltration of the exocrine glands [6-8]. Although its etiology remains incompletely understood, the disease is thought to result from immune-mediated mechanisms, with genetic and environmental factors contributing to its pathogenesis. Inflammatory infiltration and cytokine-mediated impairment of acinar cell function contribute to secretory dysfunction [8].

Sjögren syndrome predominantly affects women, accounting for approximately 90% of cases, and is most frequently diagnosed around the age of 50 years, although it may also occur in young adults between 20 and 30 years of age. pSS is characterized by salivary and lacrimal gland dysfunction in the absence of another autoimmune disease, whereas secondary Sjögren syndrome occurs in association with other autoimmune conditions. The disease affects both major and minor salivary glands, leading to acinar atrophy and, in some cases, salivary gland enlargement [6,9,10].

Reduced salivary flow contributes to an increased prevalence of *Candida *species, particularly *Candida albicans*, and is associated with a range of oral manifestations, including candidiasis, oral ulcerations, gingival inflammation, dry and erythematous mucosa, burning mouth symptoms, and halitosis. Patients may also present with cutaneous dryness [9-11]. Studies have also demonstrated an increased risk of carious lesions in atypical locations, including root surfaces and subgingival areas, together with a higher rate of failure and deterioration of dental restorations, reflecting the reduced longevity of restorative materials in the severely xerostomic oral environment [10-13]. Beyond its oral manifestations, Sjögren syndrome may significantly impair quality of life through chronic fatigue and poor sleep quality. Clinical presentation may vary with age, and major salivary gland inflammation may occur in younger patients. pSS is also associated with an increased risk of non-Hodgkin lymphoma [9,10,14]. Objective assessment of salivary flow is important in distinguishing subjective xerostomia from hyposalivation. Early recognition is therefore essential to improve patients’ quality of life and reduce the proliferation of pathogenic microorganisms within the oral cavity [8,15]. The broad clinical presentation and potential diagnostic delay emphasize the importance of early recognition. Diagnosis relies on the assessment of ocular dryness, salivary gland involvement, and autoimmune activity [16].

Impact of Sjögren syndrome on periodontal tissues

pSS may contribute to periodontal susceptibility through both salivary dysfunction and immune-mediated inflammatory mechanisms. Under physiological conditions, saliva plays a fundamental role in maintaining oral and periodontal homeostasis. It provides mechanical clearance, lubrication, buffering, mineral homeostasis, and antimicrobial protection, thereby helping regulate the oral microbiota and maintain tissue integrity. In pSS, both the quantity and composition of saliva are altered, impairing these protective functions and creating an oral environment that favors microbial dysbiosis and persistent inflammation. Reduced salivary flow and alterations in salivary composition further impair these protective functions and facilitate plaque accumulation on tooth surfaces [17,18]. The persistence and maturation of dental biofilm may subsequently promote gingival inflammation and deterioration of periodontal parameters [17,19].

In addition, Sjögren syndrome is associated with local immune alterations, including lymphocytic infiltration around the salivary ducts, changes in gingival microcirculation, increased inflammatory cytokine production, and higher serum concentrations of antibodies against periodontal pathogens such as *Aggregatibacter actinomycetemcomitans* and *Porphyromonas gingivalis *[17]. Among these inflammatory mediators, IL-6 appears to play a central role. Salivary IL-6 levels are consistently elevated in patients with pSS and have been associated with hyposalivation, positive minor salivary gland histology, and disease severity [20,21]. Furthermore, IL-6 is a key mediator of periodontal inflammation and bone resorption, suggesting that it may represent an important biological link between Sjögren syndrome and periodontal tissue destruction [19,22]. In addition to IL-6, other salivary inflammatory biomarkers, including calprotectin, have been reported to be elevated in patients with pSS and correlate with disease severity or salivary inflammation [21].

Recent proteomic and microRNA studies have also identified additional salivary biomarker candidates associated with the altered inflammatory environment in pSS, although their contribution to periodontal breakdown remains unclear and their clinical application is currently limited by methodological variability and patient heterogeneity [18,21].

Nevertheless, despite these biologically plausible mechanisms, the pathophysiological relationship between Sjögren syndrome and periodontal disease remains incompletely understood, and a direct causal association has not yet been established [17]. Clinical studies investigating the periodontal status of patients with pSS have produced heterogeneous findings. Several studies have reported increased plaque accumulation, gingival bleeding, probing pocket depth, clinical attachment loss, gingival crevicular fluid volume, and tooth loss compared with healthy controls, suggesting greater periodontal impairment in affected patients [17,19,23]. Reduced salivary flow is considered a major contributing factor, as it favors plaque accumulation and subsequent periodontal inflammation [17,19]. However, not all periodontal parameters appear to be consistently affected. In one study, patients with pSS exhibited significantly greater clinical attachment loss and gingival crevicular fluid despite presenting plaque and gingival indices comparable to those of healthy controls, indicating that mechanisms other than biofilm accumulation may contribute to periodontal breakdown [23]. Moreover, a higher prevalence of tooth loss and edentulism has been reported among patients with Sjögren syndrome, further supporting the potential impact of the disease on long-term periodontal health [17].

Overall, pSS may increase susceptibility to periodontal inflammation through the combined effects of hyposalivation and immune dysregulation; however, the available evidence remains inconclusive because of heterogeneity in study design, patient characteristics, disease severity, and periodontal assessment methods [17]. These findings emphasize the importance of regular periodontal monitoring and preventive care in patients with pSS while highlighting the need for well-designed prospective studies to clarify this association [17,19]. These findings also raise the question of whether similar mechanisms may influence peri-implant tissue health and long-term implant outcomes.

Peri-implant health in patients with pSS

Dental implant therapy in patients with Sjögren syndrome generally shows favorable short- to medium-term outcomes, with survival rates broadly comparable to those reported in individuals without the disease. Cohort and prospective studies have consistently reported favorable implant outcomes in patients with pSS, with survival rates ranging from 97% after a median follow-up of 46 months to 100% after five years. Moreover, implant performance was found to be comparable to that of matched healthy controls during follow-up periods of up to five years [24-26]. A case series combined with a systematic review reported a pooled implant failure rate of 4.1%, with a lower failure rate in primary than in secondary Sjögren syndrome, while another systematic review found an average survival rate of approximately 93.7% over nearly five years [3,27]. Nevertheless, the available evidence remains heterogeneous, and an overview of systematic reviews found that most published reviews were of low or critically low methodological quality, limiting the reliability of pooled survival estimates [28].

Although implant survival appears favorable, patients with Sjögren syndrome may be more susceptible to peri-implant soft tissue inflammation. Reduced salivary secretion and impaired oral self-clearance facilitate the retention of debris and plaque on implant surfaces, exposing the peri-implant mucosa to repeated inflammatory challenges [24,26,27]. Clinical studies have therefore reported slightly higher gingival indices, bleeding on probing, and probing depths in patients with Sjögren syndrome than in healthy controls, even when plaque scores were similar [24-26]. Peri-implant mucositis appears to be the most frequently reported biological complication and has been observed in up to 94% of patients in one cohort, compared with 62% of healthy controls [1,27]. However, despite the high prevalence of mucosal inflammation, peri-implantitis rates have generally remained limited and comparable to those of healthy controls, suggesting that soft tissue inflammation does not necessarily translate into progressive peri-implant bone destruction during short- or medium-term follow-up [1,24,25].

Several studies have reported marginal bone levels within ranges considered acceptable for healthy implant populations, with no significant differences in marginal bone loss between patients with pSS and matched controls over five years [24,25,27]. A single case report similarly documented the absence of peri-implant bone resorption or soft tissue inflammation three years after implant rehabilitation [2]. However, some evidence suggests that patients with Sjögren syndrome may experience greater marginal bone loss than the general population, particularly in mixed cohorts including both primary and secondary forms of the disease [3]. Interpretation remains difficult because many studies lacked standardized baseline radiographs, used panoramic rather than standardized intraoral imaging, or included small and heterogeneous samples [26,27,29].

The distinction between primary and secondary Sjögren syndrome may also influence peri-implant outcomes. pSS has been associated with lower implant failure rates than secondary Sjögren syndrome, possibly because patients with secondary disease may also have rheumatoid arthritis, systemic lupus erythematosus, reduced manual dexterity, or exposure to immunosuppressive treatment, all of which may complicate healing and plaque control [3,25,26,30]. Nevertheless, current evidence does not consistently demonstrate significant differences in peri-implant outcomes between primary and secondary Sjögren syndrome, and many studies do not report results separately according to disease subtype. This lack of stratification represents a major limitation and prevents firm conclusions regarding the independent effect of pSS on implant prognosis [27,28].

Maintenance is particularly important for preserving peri-implant health. Regular recall visits, shortened professional hygiene intervals, prosthetic designs that facilitate plaque control, and individualized maintenance programs are particularly important because xerostomia promotes plaque adherence and, in some patients, associated arthritis may reduce manual dexterity [24,27,30]. One report described peri-implant mucositis in a patient with secondary Sjögren syndrome that resolved after implementation of an individualized maintenance program [30]. Close monitoring and rigorous professional and home care may therefore help maintain periodontal and peri-implant health [29].

Implant-supported rehabilitation may improve oral health-related quality of life, comfort, and satisfaction, although persistent oral dryness may continue to affect chewing ability and oral function [3,25,26,29,31]. Overall, current evidence supports the feasibility of implant therapy in patients with pSS when peri-implant inflammation is closely monitored and long-term maintenance is provided. However, the predominance of small observational studies, short follow-up periods, heterogeneous patient populations, and limited standardization means that long-term risks, particularly the progression from peri-implant mucositis to peri-implantitis, cannot yet be excluded [1,25,27-31].

These findings are consistent with the clinical course observed in our patient, who presented with peri-implant mucosal inflammation at the two-year follow-up without radiographic evidence of marginal bone loss around the implants placed at sites 14 and 16. Although the implants remained clinically and radiographically stable, the presence of peri-implant mucosal inflammation highlights the potential susceptibility of patients with pSS to peri-implant soft tissue inflammation. This finding further supports the importance of regular maintenance and long-term follow-up in preserving peri-implant health.

Interpretation of the findings

The present case illustrates that successful implant rehabilitation is achievable in patients with pSS despite the chronic inflammatory environment associated with the disease. In the present patient, peri-implant mucosal inflammation developed despite clinically stable implants and the absence of radiographic marginal bone loss at the two-year follow-up. This combination of findings suggests that, although patients with pSS may be more susceptible to peri-implant soft tissue inflammation, peri-implant mucosal inflammation and implant survival should be interpreted as distinct clinical outcomes. Persistent hyposalivation may reduce the natural cleansing capacity of saliva, promoting plaque retention around implant surfaces, while the chronic immune dysregulation characteristic of pSS may amplify the local inflammatory response. These mechanisms may be relevant to the development of peri-implant soft tissue inflammation [1,25-27,29].

An important finding in this case was the absence of radiographic marginal bone loss despite the presence of mucosal inflammation. This observation is consistent with current evidence suggesting that peri-implant mucosal inflammation represents a reversible inflammatory condition affecting the peri-implant soft tissues and does not inevitably progress to peri-implantitis. Nevertheless, because patients with pSS exhibit persistent xerostomia and impaired oral clearance, the long-term evolution of peri-implant inflammation remains uncertain, and progression to peri-implantitis cannot be excluded. Although limited to a single patient with a two-year follow-up, this case contributes to the clinical literature describing implant therapy as a treatment option for patients with pSS, while also highlighting that peri-implant soft tissue inflammation may represent the principal biological challenge in this population. At the same time, it emphasizes that implant success should not be evaluated solely by survival rates but also by careful assessment of peri-implant soft tissue health. Long-term clinical and radiographic follow-up, combined with individualized supportive maintenance, remains important to preserve peri-implant health and detect early biological complications before irreversible tissue destruction occurs [1,3,24,25,28,30].

The present case has several limitations that should be considered when interpreting its findings. First, this report describes the clinical course of a single patient, which limits the generalizability of the observations. Second, the follow-up period of two years does not allow conclusions regarding the long-term progression of peri-implant mucosal inflammation or the future development of peri-implantitis. Third, the absence of quantitative implant stability measurements using resonance frequency analysis and three-dimensional cone-beam CT assessment during follow-up limits the objective assessment of implant stability and marginal bone-level changes over time. The radiographic assessment was based on available periapical radiographs, and the absence of standardized angulation and positioning represents a limitation when assessing changes in marginal bone levels. Fourth, although clinical and radiographic examinations demonstrated stable peri-implant bone levels, no microbiological analyses, quantitative salivary flow measurements, or biomarker assessments were performed to further characterize the biological mechanisms underlying the observed peri-implant inflammation. Finally, because peri-implant mucosal conditions before the diagnosis of pSS were not documented using standardized baseline indices, the precise temporal relationship between disease onset and peri-implant inflammation cannot be fully established. Future prospective studies with larger cohorts, standardized peri-implant assessments, and longer follow-up periods are needed to clarify the long-term impact of pSS on peri-implant tissue health.

Diagnostic implications for dental practitioners

Serological testing and minor labial salivary gland biopsy remain the two principal diagnostic tools for distinguishing Sjögren syndrome from other causes of xerostomia. However, because a substantial proportion of patients are seronegative, the absence of anti-SSA and anti-SSB autoantibodies does not exclude the diagnosis. In patients presenting with persistent sicca symptoms, negative serological findings should therefore prompt further investigation, particularly by minor salivary gland biopsy. This emphasizes the importance of evaluating Sjögren-associated xerostomia beyond serological testing alone [16,32].

Minor salivary gland biopsy plays a pivotal role in the diagnostic pathway when clinical suspicion remains high despite negative serological findings. The procedure involves obtaining four to six minor salivary glands from the lower lip for histopathological evaluation, which typically demonstrates focal lymphocytic sialadenitis, the hallmark lesion of Sjögren syndrome. In the present case, the biopsy was reported as Chisholm and Mason grade 3 and was considered compatible with Sjögren syndrome in the clinical context. Establishing an early diagnosis allows timely referral to a multidisciplinary team and facilitates appropriate medical management and monitoring for potential systemic complications, including lymphoma [16,32-34]. Beyond its diagnostic value, the present case highlights the pivotal role of dental practitioners in both the early recognition and long-term management of pSS. Dentists are often the first healthcare professionals to evaluate patients presenting with persistent xerostomia, xerophthalmia, recurrent caries, unexplained periodontal inflammation, or peri-implant soft tissue changes. Recognition of these clinical findings should prompt consideration of Sjögren syndrome as part of the differential diagnosis and early referral for definitive investigation and multidisciplinary management [35].

As implant therapy becomes increasingly common in patients with pSS, clinicians should recognize that implant success extends beyond implant survival alone. The present case demonstrates that peri-implant mucosal inflammation may develop despite stable osseointegration and the absence of radiographic marginal bone loss. Consequently, careful evaluation of peri-implant soft tissues should complement routine radiographic assessment during maintenance visits. Individualized supportive periodontal therapy, meticulous oral hygiene reinforcement, and regular clinical and radiographic follow-up are important to preserve peri-implant health and reduce the risk of progression from peri-implant mucositis to peri-implantitis.

The present case illustrates that successful implant rehabilitation in patients with pSS depends not only on achieving osseointegration but also on maintaining peri-implant tissue health through early diagnosis, multidisciplinary collaboration, and individualized long-term supportive care. Increased awareness of the oral manifestations of Sjögren syndrome among dental practitioners may facilitate earlier diagnosis, improve long-term implant maintenance, and ultimately enhance both oral and systemic patient outcomes.

## Conclusions

This case report describes the two-year clinical and radiographic follow-up of dental implants in a patient with pSS. During the documented follow-up period, peri-implant mucosal inflammation was observed, while no clinical or radiographic evidence of peri-implantitis or progressive marginal bone loss was identified. These findings represent the clinical course observed in this individual patient.

The oral manifestations of pSS, including hyposalivation, altered salivary composition, and immune dysregulation, may increase susceptibility to periodontal and peri-implant inflammation even in the absence of peri-implant bone loss. Consequently, regular supportive periodontal and peri-implant maintenance, meticulous plaque control, and close clinical monitoring are important to preserve peri-implant tissue health in these patients. Finally, this case highlights the pivotal role of dentists in the early recognition of persistent xerostomia and xerophthalmia. Prompt referral for appropriate diagnostic investigations and multidisciplinary management may facilitate earlier diagnosis of pSS, improve patient outcomes, and optimize the long-term prognosis of implant-supported rehabilitation. Further prospective studies with larger cohorts and longer follow-up periods are required to better clarify the influence of pSS on peri-implant tissue health and long-term implant outcomes.
